# Modulation of Dorsolateral Prefrontal Cortex Glutamate/Glutamine Levels Following Repetitive Transcranial Magnetic Stimulation in Young Adults With Autism

**DOI:** 10.3389/fnins.2021.711542

**Published:** 2021-10-06

**Authors:** Iska Moxon-Emre, Zafiris J. Daskalakis, Daniel M. Blumberger, Paul E. Croarkin, Rachael E. Lyon, Natalie J. Forde, Hideaki Tani, Peter Truong, Meng-Chuan Lai, Pushpal Desarkar, Napapon Sailasuta, Peter Szatmari, Stephanie H. Ameis

**Affiliations:** ^1^Cundill Centre for Child and Youth Depression, The Margaret and Wallace McCain Centre for Child, Youth & Family Mental Health, Campbell Family Mental Health Research Institute, Centre for Addiction and Mental Health, Toronto, ON, Canada; ^2^Temerty Centre for Therapeutic Brain Intervention, Campbell Family Mental Health Research Institute, Centre for Addiction and Mental Health, Toronto, ON, Canada; ^3^Department of Psychiatry, Temerty Faculty of Medicine, University of Toronto, Toronto, ON, Canada; ^4^Division of Child and Adolescent Psychiatry, Department of Psychiatry and Psychology, Mayo Clinic, Rochester, MN, United States; ^5^Radboud University Medical Centre, Donders Institute for Brain, Cognition and Behaviour, Nijmegen, Netherlands; ^6^Research Imaging Centre, Centre for Addiction and Mental Health, Toronto, ON, Canada; ^7^Department of Psychiatry, Research Institute, The Hospital for Sick Children, Toronto, ON, Canada; ^8^Department of Psychology, University of Toronto, Toronto, ON, Canada

**Keywords:** autism spectrum disorder, repetitive transcranial magnetic simulation, dorsolateral prefrontal cortex, magnetic resonance spectography, Glx, GABA, MEGA-PRESS

## Abstract

Altered excitatory and inhibitory neurotransmission has been implicated in autism spectrum disorder (ASD). Interventions using repetitive transcranial magnetic stimulation (rTMS) to enhance or inhibit cortical excitability are under study for various targets, though the mechanistic effects of rTMS have yet to be examined in ASD. Here, we examined whether an excitatory rTMS treatment course modulates glutamatergic (Glx) or γ-aminobutyric acid (GABA) metabolite levels in emerging adults with ASD. Twenty-eight participants with ASD and executive function impairment [23.3 ± 4.69 years; seven-female] underwent two magnetic resonance spectroscopy (MRS) scans of the left dorsolateral prefrontal cortex (DLPFC). MRS scans were acquired before and after participants with ASD were randomized to receive a 20-session course of active or sham rTMS to the DLPFC. Baseline MRS data was available for 19 typically developing controls [23.8 ± 4.47 years; six-female]. Metabolite levels for Glx and GABA+ were compared between ASD and control groups, at baseline, and metabolite level change, pre-to-post-rTMS treatment, was compared in ASD participants that underwent active vs. sham rTMS. Absolute change in Glx was greater in the active vs. sham-rTMS group [*F*_(1,__19)_ = 6.54, *p* = 0.02], though the absolute change in GABA+ did not differ between groups. We also examined how baseline metabolite levels related to pre/post-rTMS metabolite level change, in the active vs. sham groups. rTMS group moderated the relation between baseline Glx and pre-to-post-rTMS Glx change, such that baseline Glx predicted Glx change in the active-rTMS group only [*b* = 1.52, *SE* = 0.32, *t*_(18)_ = 4.74, *p* < 0.001]; Glx levels increased when baseline levels were lower, and decreased when baseline levels were higher. Our results indicate that an interventional course of excitatory rTMS to the DLPFC may modulate local Glx levels in emerging adults with ASD, and align with prior reports of glutamatergic alterations following rTMS. Interventional studies that track glutamatergic markers may provide mechanistic insights into the therapeutic potential of rTMS in ASD.

**Clinical Trial Registration:** Clinicaltrials.gov (ID: NCT02311751), https://clinicaltrials.gov/ct2/show/NCT02311751?term=ameis&rank=1; NCT02311751.

## Introduction

Autism spectrum disorder (ASD) is a neurodevelopmental disorder characterized by complex phenotypic and neurobiological heterogeneity. A number of studies point to the possible convergence of altered excitatory glutamatergic and inhibitory γ-aminobutyric acid (GABA) mediated neurotransmission in ASD (Ford and Crewther, 2016; Ajram et al., 2019). Glutamate and GABA are fundamentally important for the development of neuronal circuitry, and maintenance of cognition and behavior (Lujan et al., 2005). Importantly, glutamate and GABA are not independent neural chemicals; within GABAergic interneurons, glutamine is synthesized into glutamate, which is subsequently synthesized into GABA by the glutamate decarboxylase (GAD) enzyme (Bak et al., 2006). The rate-limiting GAD enzyme may be altered in ASD (Yip et al., 2007). Thus, relative levels of glutamate and GABA may differ among individuals with ASD, as glutamate, glutamine and GABA are continually in flux.

Magnetic resonance spectroscopy (MRS) is one of the few non-invasive techniques able to probe biochemistry in the human brain through measurement of metabolites associated with neurophysiological processes (Rae, 2014). Though findings are mixed, a number of MRS studies report altered GABA and Glx (glutamate + glutamine) levels, across various brain regions, in participants with ASD compared to typically developing controls (TDC) (Ford and Crewther, 2016; Ajram et al., 2019). Within prefrontal regions, lower GABA and/or Glx levels have been detected in samples of children with ASD vs. TDC (Harada et al., 2011; Kubas et al., 2012). There are comparatively fewer studies that evaluate GABA and Glx levels in autistic adults. Both lower (Bernardi et al., 2011; Horder et al., 2013, 2018; Tebartz van Elst et al., 2014) and higher Glx (Page et al., 2006; Brown et al., 2013) levels have been reported in available studies of adults with ASD vs. TDC, though no GABA differences have been found. Of note, studies that include samples spanning across the child, youth and young adult age ranges have not shown differences in GABA or Glx levels in ASD vs. TDC (Ajram et al., 2019), suggesting that age may have influenced prior neurometabolite findings.

Various pharmacological agents that affect glutamate/GABA signaling are currently under study as interventions in ASD [e.g., Memantine, riluzole, arbaclofen (Lai et al., 2020), cannabinoid compounds (Pretzsch et al., 2019a,b)]. Identifying non-invasive approaches that modulate this neurotransmitter pathway, and metrics that can track successful modulation, represent important steps to development of biomedical interventions in this area. Repetitive transcranial magnetic stimulation (rTMS) is a non-invasive interventional tool that involves stimulating the cortex with trains of magnetic pulses (George et al., 2009). Although TMS studies implicate aberrant cortical plasticity in ASD (Oberman et al., 2012; Oberman et al., 2016), we are not aware of any study that has examined rTMS effects on neurometabolite levels in this population.

Repetitive transcranial magnetic stimulation may drive changes in excitatory and inhibitory tone through a variety of mechanisms, such as changes to glutamatergic synapses, GABAergic neurons, brain derived neurotrophic factor, or promotion of neurogenesis (Dayan et al., 2013; Polania et al., 2018). However, the neurobiological effect of rTMS may depend upon the individual characteristics of the brain undergoing stimulation (Silvanto et al., 2008). For example, in major depressive disorder (MDD), rTMS is thought to restore normative brain function through facilitating the re-emergence of intrinsic cerebral rhythms (Leuchter et al., 2013). Further, a study in a non-clinical sample suggested that although rTMS paradigms can increase cortical inhibition, the extent of change may depend on baseline inhibition (i.e., larger increases in individuals with lower baseline inhibition found) (Daskalakis et al., 2006).

The potential utility of rTMS in the treatment of neuropsychiatric conditions stems, in part, from its ability to enhance or inhibit cortical excitability in targeted brain regions. Preliminary evidence suggests that rTMS to the dorsolateral prefrontal cortex (DLPFC) may have value as an interventional tool to alter repetitive or stereotyped behaviors, social functioning (Barahona-Correa et al., 2018), depressive symptoms (Gwynette et al., 2020), or executive functioning (Sokhadze et al., 2014) in ASD.

Our recent randomized double-blind sham-controlled pilot trial of 20 sessions of 20 Hz (excitatory) rTMS to bilateral DLPFC, tested the feasibility and preliminary efficacy of rTMS for the treatment of executive function deficits in emerging adults with ASD. Stimulation parameters for our trial targeting executive function impairment were chosen according to the best available evidence for improving cognitive function in clinical populations at the time of trial design. For example, a systematic review that assessed the potential for rTMS to improve cognitive outcome across various clinical populations found that a course of high frequency stimulation to DLPFC was most promising for improving executive function (Guse et al., 2010). Preliminary studies in ASD had also suggested that bilateral DLPFC stimulation at 90% resting motor threshold (RMT) may improve performance on cognitive domains (Sokhadze et al., 2014) and that a course of high frequency bilateral prefrontal cortex stimulation was feasibly implemented in an ASD sample at 90% RMT (Enticott et al., 2014). Our pilot rTMS clinical trial in ASD used the exact same stimulation protocol (20 sessions of 20 Hz rTMS to bilateral DLPFC at 90% RMT) as a prior positive clinical trial that tested the efficacy and feasibility of rTMS to improve working memory in schizophrenia (Barr et al., 2013). Individuals with ASD are predisposed to seizures and often take medications similar to individuals with schizophrenia. Therefore, we specifically chose to model our pilot clinical trial parameters to be consistent with a protocol that had improved working memory deficits (our main clinical outcome measure of the trial), and that was safely and feasibly implemented in a complex clinical population (Barr et al., 2013). Additionally, we used target site and intensity parameters that had been safely implemented in prior published rTMS studies in ASD samples (Enticott et al., 2014; Sokhadze et al., 2014). Though we did not find significant differences in executive function performance following active versus sham treatment across our clinical trial sample, executive functioning improved following active rTMS in the subset of participants in our sample with more pronounced baseline functional impairments (Ameis et al., 2020).

In the present study, we analyzed available ^1^H MRS data measuring GABA+ (GABA+ macromolecules) and Glx levels in individuals with ASD that participated in our 4-week pilot clinical trial studying the effects of rTMS to DLPFC on executive function deficits (Ameis et al., 2020). MRS data was collected as part of the trial to explore changes in inhibitory and excitatory neurotransmission following rTMS. No *a priori* hypotheses for MRS data were registered prior to the clinical trial. Owing to the mixed evidence for neurometabolite alterations in ASD, we first examined whether GABA+ or Glx levels within the DLPFC differed in ASD vs. age-matched TDCs, at baseline. However, the primary objective of our study was to test the hypothesis that GABA+ and Glx levels within the DLPFC would change following active vs. sham rTMS in participants with ASD. Based on the potential for state-dependent effects following rTMS (Daskalakis et al., 2006; Kearney-Ramos et al., 2019), we also explored whether the direction of metabolite level change was influenced by baseline metabolite levels.

## Materials and Methods

### Participants

#### Baseline Autism Spectrum Disorder Group

Forty participants with ASD, characterized previously (Ameis et al., 2017, 2020), were recruited from the Centre for Addiction and Mental Health (CAMH, Toronto, Canada), local community clinics, and advertisements (local and online). Among the 40 participants that completed the rTMS clinical trial, 33 participants underwent MRS at least once, and 28 had useable baseline scans (see Supplementary Table 1 and Table 1). Characteristics of the participants with and without useable MRS scans are provided in Supplementary Table 2. This study was part of a pilot clinical trial designed to investigate the potential of rTMS as an intervention for executive function deficits in ASD. The study was approved by the CAMH research ethics board (REB; protocol #119-2013) and registered with Clinicaltrials.gov (ID: NCT02311751). The inclusion/exclusion criteria for trial participants with ASD were described previously (Ameis et al., 2017, 2020). Briefly, participants with ASD were included if they were aged 16-35 years, fluent in English, had a DSM-IV-TR diagnosis of autistic disorder, Asperger’s disorder, or pervasive developmental disorder-not otherwise specified (PDD-NOS), or a DSM-5 diagnosis of ASD. Prior clinical diagnoses were confirmed on clinical interview and using the Autism Diagnostic Observation Schedule-2 (ADOS-2), Module 4 (administered by a trained child and youth psychiatrist, SHA) (Lord et al., 2000). Capacity to consent, clinical stability, an IQ ≥ 70 on the General Abilities Index (GAI) from the Wechsler Adult Intelligence Scale-Fourth Edition (WAIS-IV) (Benson et al., 2010), and a *T* score > 65 on any subscale of the Behavior Rating Inventory of Executive Function (BRIEF)-self report version (Gioia et al., 2002), indicating clinically significant impairment in executive functioning, were also required for inclusion. Adaptive functioning was assessed with the Vineland Adaptive Behavior Scale-II (VABS-II) (Sparrow and Cicchetti, 1985). Co-occurring mental health conditions were assessed using the Mini International Neuropsychiatric Interview (MINI) (Sheehan et al., 1998). Participants were excluded if they had prior major medical or neurological illnesses, were taking anticonvulsants or benzodiazepines (≥2 mg lorazepam equivalent), were pregnant or had potential for pregnancy, had history of substance use/dependence within the last 6 months or a positive urine toxicology screen, had history of rTMS treatment, were unable to commit to the rTMS protocol, or unable to consent to participation. No changes in psychotropic medication were permitted within 4 weeks of randomization to the end of treatment. Psychiatric comorbidities, and psychotropic medications are detailed in Supplementary Table 3.

**TABLE 1:** Characteristics of the baseline sample (TDC vs. all ASD participants).

	**TDC (*n* = 19)**	**ASD (*n* = 28)**	**Test**	***p*-value**
**Age**				
Mean (*SD*)	23.8 (4.47)	23.3 (4.69)	*t*_(__40__)_ = 0.40	0.69
Median [Min, Max]	23.0 [16.0, 34.0]	22.0 [16.0, 33.0]		
**Sex**				
Number of males (%)	13 (68.4%)	21 (75.0%)	*X*^2^ = 0.03	0.87
Psychotropic Medication^*a*,^*				
Number of participants on (%)	0 (0%)	17 (60.7%)	Fisher’s Exact	**<0.001**
**MINI^*a*^**				
**Comorbidity**				
Number of participants (%)	0 (0%)	16 (57.1%)	Fisher’s Exact	**<0.001**
**Depression – current (2 weeks)**				
Number of participants (%)	0 (0%)	7 (25.0%)	Fisher’s Exact	**0.03**
**Depression – recurrent**				
Number of participants (%)	0 (0%)	2 (7.1%)	Fisher’s Exact	0.15
**Years of Education^*a*^**				
Mean (*SD*)	15.4 (2.31)	14.2 (3.01)	*t*_(__43__)_ = 1.56	0.13
Median [Min, Max]	16.0 [10.0, 19.0]	13.5 [10.0, 22.0]		
**IQ – General Abilities Index^*b*^**				
Mean (*SD*)	111 (9.37)	112 (17.8)	*t*_(__42__)_ = –0.23	0.82
Median [Min, Max]	111 [94.0, 136]	111 [77.0, 141]		
**BRIEF Metacognition Index^*a*^**				
Mean (*SD*)	45.7 (6.88)	70.6 (8.21)	*U* = 1.5	**<0.001**
Median [Min, Max]	45.0 [36.0, 59.0]	68.5 [59.0, 84.0]		
**BRIEF Global Composite^*a*^**				
Mean (*SD*)	43.5 (5.89)	68.0 (8.18)	*t*_(__43__)_ = –11.81	**<0.001**
Median [Min, Max]	43.5 [35.0, 55.0]	66.5 [52.0, 86.0]		
**Adaptive Functioning Composite**				
Mean (*SD*)	–	75.5 (9.88)	–	–
Median [Min, Max]	–	74.5 [58.0, 104]		
**WM Fraction**				
Mean (*SD*)	0.415 (0.0663)	0.395 (0.0841)	*F*_(__1,__44__)_ = 0.63	0.43
Median [Min, Max]	0.407 [0.311, 0.528]	0.397 [0.222, 0.585]		
**GM Fraction**				
Mean (*SD*)	0.459 (0.0392)	0.461 (0.0512)	*F*_(__1,__44__)_ < 0.001	0.98
Median [Min, Max]	0.467 [0.373, 0.507]	0.466 [0.340, 0.557]		
**CSF Fraction**				
Mean (*SD*)	0.116 (0.0341)	0.129 (0.0300)	*F*_(__1,__44__)_ = 1.80	0.19
Median [Min, Max]	0.123 [0.0567, 0.163]	0.128 [0.0742, 0.208]		
**GABA+**				
Mean (*SD*)	0.182 (0.0219)	0.175 (0.0292)	*F*_(__1,__44__)_ = 0.59	0.45
Median [Min, Max]	0.181 [0.134, 0.230]	0.168 [0.133, 0.253]		
**Glx**				
Mean (*SD*)	0.121 (0.0172)	0.120 (0.0208)	*F*_(__1,__44__)_ = 0.03	0.86
Median [Min, Max]	0.123 [0.0912, 0.157]	0.116 [0.0846, 0.166]		
**GABA+/Glx ratio**				
Mean (*SD*)	1.52 (0.256)	1.48 (0.198)	*F*_(__1,__44__)_ = 0.44	0.51
Median [Min, Max]	1.46 [1.24, 2.37]	1.46 [1.21, 1.92]		

*^*a*^Data was missing from 1 control participant.*

*^*b*^Data was missing from 2 control participants.*

*Psychotropic medication is detailed in Supplementary Table 3.

*Age, years of education, IQ and BRIEF scores were compared between groups using Welch’s *t*-tests or Mann–Whitney’s *U* tests when data was not normally distributed. Sex (which was determined based on participant self report) was compared with a Chi-Square Test. Fisher’s Exact tests were used when values in any cell were <5. Statistical tests for biological measures (i.e., tissue fractions and metabolite levels) represent groups comparisons, covarying for age.*

*ASD, autism spectrum disorder; BRIEF, behavior rating inventory of executive function; TDC, typically developing controls; MINI, Mini International Neuropsychiatric Interview; WM, white matter; GM, gray matter; CSF, cerebrospinal fluid. Bold values denote statistical significance at p < 0.05.*

#### Baseline Comparison/Control Group

Twenty age- and sex-matched TDCs were recruited from local and online advertisements; all participants underwent MRS once, and 19 had useable baseline scans (Supplementary Table 1 and Table 1). Control participants were included if they were aged 16–35 years, fluent in English, and had capacity to consent. TDC participants were excluded if they had a history of substance use/dependence within the last 6 months, a positive urine toxicology screen, any major medical or neurological illness, a diagnosed learning disorder, an IQ < 70, were pregnant, or if they were found to have a psychiatric diagnosis during the MINI assessment. Participant characteristics are detailed in Table 1.

#### Repetitive Transcranial Magnetic Stimulation Intervention Group

Demographic and clinical characteristics for the 28 participants with ASD with available MRS data that were treated with active (*n* = 16) vs. sham (*n* = 12) rTMS, are provided in Table 2. The breakdown of participants with MRS scans, within each treatment group, is detailed in Supplementary Table 1. Of note, the number of participants with both pre- and post-rTMS scans was slightly smaller (active: *n* = 12; sham: *n* = 10) (Supplementary Table 4). Both cohorts were included, for reasons detailed in the statistical analyses section below.

**TABLE 2:** Characteristics of the clinical trial sample (participants with ASD in the active vs. sham rTMS groups).

	**Active rTMS**		**Sham rTMS**			
	**(*n* = 16)**		**(*n* = 12)**		**Test**	***p*-value**
**Age**						
Mean (*SD*)	23.1 (4.66)		23.4 (4.93)		*t*_(__23__)_ = –0.16	0.88
Median [Min, Max]	21.5 [16.0, 33.0]		23.5 [16.0, 31.0]			
**Sex**						
Number of males (%)	13 (81.2%)		8 (66.7%)		Fisher’s Exact	0.42
Psychotropic Medication*						
Number of participants on (%)	12 (75.0%)		5 (41.7%)		Fisher’s Exact	**<0.001**
**MINI**						
**Comorbidity**						
Number of participants (%)	10 (62.5%)		6 (50.0%)		Fisher’s Exact	**0.04**
**Depression – current (2 weeks)**						
Number of participants (%)	2 (12.5%)		5 (41.7%)		Fisher’s Exact	**<0.001**
**Depression – recurrent**						
Number of participants (%)	0 (0%)		2 (16.7%)		Fisher’s Exact	**0.004**
**Years of Education**						
Mean (*SD*)	14.9 (3.11)		13.3 (2.70)		*U* = 129	0.13
Median [Min, Max]	15.0 [10.0, 22.0]		12.0 [10.0, 18.0]			
**IQ – General Abilities Index**						
Mean (*SD*)	112 (19.5)		112 (16.1)		*t*_(__26__)_ = 0.009	0.99
Median [Min, Max]	114 [77.0, 140]		111 [92.0, 141]			
**BRIEF Metacognition Index**						
Mean (SD)	70.8 (7.63)		70.3 (9.27)		*t*_(__21__)_ = 0.15	0.89
Median [Min, Max]	69.5 [59.0, 84.0]		68.0 [59.0, 84.0]			
**BRIEF Global Composite**						
Mean (*SD*)	67.1 (7.99)		69.3 (8.61)		*U* = 87.5	0.71
Median [Min, Max]	66.5 [52.0, 86.0]		65.5 [61.0, 83.0]			
**Adaptive Functioning Composite**						
Mean (*SD*)	75.8 (8.13)		75.2 (12.2)		*t*_(__18__)_ = 0.16	0.88
Median [Min, Max]	75.5 [61.0, 89.0]		72.0 [58.0, 104]			

	**Pre**	**Post**	**Pre**	**Post**		
	**(*n* = 16)**	**(*n* = 12)**	**(*n* = 12)**	**(*n* = 12)**		

**WM fraction**						
Mean (*SD*)	0.399 (0.0857)	0.432 (0.105)	0.390 (0.0853)	0.406 (0.102)	*F*_(__1,__25__)_ = 0.25	0.62
Median [Min, Max]	0.397 [0.222, 0.534]	0.423 [0.310, 0.600]	0.389 [0.268, 0.585]	0.383 [0.270, 0.588]		
**GM fraction**						
Mean (*SD*)	0.460 (0.0527)	0.438 (0.0729)	0.462 (0.0515)	0.453 (0.0673)	*F*_(__1,__25__)_ = 0.13	0.72
Median [Min, Max]	0.465 [0.370, 0.542]	0.450 [0.321, 0.542]	0.466 [0.340, 0.557]	0.452 [0.337, 0.546]		
**CSF fraction**						
Mean (*SD*)	0.128 (0.0301)	0.123 (0.0365)	0.131 (0.0311)	0.130 (0.0327)	*F*_(__1,__26__)_ = 0.16	0.69
Median [Min, Max]	0.119 [0.0948, 0.208]	0.117 [0.0794, 0.209]	0.141 [0.0742, 0.166]	0.138 [0.0754, 0.168]		
**GABA+**						
Mean (*SD*)	0.181 (0.0315)	0.193 (0.0364)	0.168 (0.0254)	0.189 (0.0502)	*F*_(__1,__26__)_ = 0.33	0.57
Median [Min, Max]	0.176 [0.141, 0.253]	0.198 [0.118, 0.239]	0.164 [0.133, 0.219]	0.170 [0.131, 0.292]		
**Glx**						
Mean (*SD*)	0.119 (0.0226)	0.130 (0.0281)	0.121 (0.0189)	0.121 (0.0254)	*F*_(__1,__25__)_ = 0.23	0.64
Median [Min, Max]	0.112 [0.0846, 0.166]	0.130 [0.0938, 0.175]	0.120 [0.0919, 0.162]	0.123 [0.0731, 0.163]		
**GABA+/Glx ratio**						
Mean (*SD*)	1.54 (0.224)	1.51 (0.297)	1.39 (0.120)	1.58 (0.287)	*F*_(__1,__25__)_ = 0.38	0.55
Median [Min, Max]	1.52 [1.21, 1.92]	1.45 [1.13, 2.25]	1.38 [1.21, 1.56]	1.48 [1.27, 2.21]		

*Psychotropic medication is detailed in Supplementary Table 3.

*Age, years of education, IQ, BRIEF, and Adaptive Functioning scores were compared between groups using Welch’s *t*-tests or Mann–Whitney’s *U* tests when data was not normally distributed. Fisher’s Exact tests were used when values in any cell were <5.*

*Statistical tests for biological measures (i.e., tissue fractions and metabolite levels) represent main effects of Group, from the 2 × 2 mixed-model ANCOVAs [group (active vs. sham rTMS) × time (pre- vs. post-rTMS)] covarying for age.*

*rTMS, repetitive transcranial magnetic stimulation; MINI, Mini International Neuropsychiatric Interview; WM, white matter; GM, gray matter; CSF, cerebrospinal fluid, BRIEF, behavior rating inventory of executive function. Bold values denote statistical significance at p < 0.05.*

### Clinical Trial Design

Participants with ASD were enrolled in a double-blind, sham-controlled trial (recruitment between November 2014 and June 2017), and were randomly allocated in a 1:1 ratio to receive active or sham rTMS treatment. Briefly, active (20 Hz, delivered at 90% RMT intensity) and sham rTMS were administered bilaterally to DLPFC (Talairach [x, y, z] = [–] 50, 30, 36), 5 days per week for 4 weeks, totaling 20 sessions, at CAMH. Stimulation was administered at 20 Hz with 25 stimulation trains of 30 stimuli each. The inter-train interval was 30 s, at equivalent stimulation parameters (Barr et al., 2013) of 750 pulses/hemisphere, totaling 1500 pulses/session. The rTMS treatment sessions lasted ∼30–45 min. 90% RMT was selected as this was the intensity used in the pilot clinical trial that our study was modeled after (Barr et al., 2013). Further, all published trials of rTMS to DLPFC in ASD available at the time of study design had stimulated at 90% RMT (Sokhadze et al., 2009, 2012, 2014; Baruth et al., 2010; Casanova et al., 2012). TDC participants were not included in the clinical trial, and thus did not receive rTMS. At the beginning of the trial, each participant was randomized to receive left or right-sided stimulation first followed by stimulation of the contralateral hemisphere (this order was maintained for all sessions). We implemented the same sham condition approach used by the study we modeled our clinical trial after; a single-wing tilt position of the coil to mimic the active rTMS condition, as this produces scalp muscle contraction with minimal direct effects on the brain (Barr et al., 2013). To test the integrity of our blinding, we asked participants following the first and last rTMS session if they believed they received active stimulation, and responses did not differ between active/sham groups (Ameis et al., 2020). Detailed clinical trial information, including sample size, randomization details (Ameis et al., 2017), and the CONSORT diagram (Ameis et al., 2020) have been published previously. Clinical trial participants underwent MRS within 1 week prior to commencing rTMS, and within 1 week following the last rTMS session.

### Magnetic Resonance Spectroscopy Data Acquisition

**FIGURE 1:**
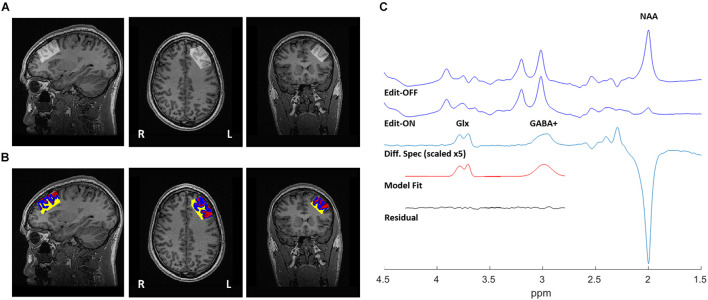
1H-MRS voxel position, tissue segmentation, and representative spectra from a single participant. **(A)** 20 mm × 40 mm × 30 mm voxel, placed in the left dorsolateral prefrontal cortex (DLPFC). **(B)** Segmentation of T1-weighted MRI, used to correct water-scaled metabolite concentrations for voxel tissue composition. Gray matter (blue), white matter (yellow), and CSF (red). **(C)** Sample output from the MEGA-PRESS sequence, with representative fits of GABA+ and Glx peaks shown in the edited spectrum.

GABA-edited proton MRS data were acquired using a MEshcher-GArwood Point RESolved Spectroscopy (MEGA-PRESS) sequence on a 3 Tesla GE MR750 (General Electric, Milwaukee, WI, United States) scanner. Each spectrum was recorded from a single 20 mm × 40 mm × 30 mm voxel, prescribed in the left DLPFC (Figure 1A). MEGA-PRESS data acquisition is single voxel method. Due to the low concentration of GABA in the brain tissue (GM GABA concentration = 1.30 + –0.36 μmol/g of brain tissue, WM GABA = 0.16 + –0.16 μmol/g tissue) (Choi et al., 2006), a large voxel is required in order to acquire enough metabolite signal for analysis. Due to imaging time-constraints in our clinical trial, we were unable to acquire MRS data from both hemispheres. Given this, we selected the left DLPFC for MRS voxel placement as left-DLPFC stimulation is more common across rTMS studies, including those with pre/post MRS data which have mainly acquired a single left hemisphere voxel (Michael et al., 2003; Luborzewski et al., 2007; Zheng et al., 2010; Croarkin et al., 2016; Dubin et al., 2016; Baeken et al., 2017; Levitt et al., 2019). Shimming was performed using GE’s manufacturer automated shimming routine (AUTOSHIM). Data acquisition parameters were: TE = 68 ms, TR = 1500 ms, 512 averages (256 editing-ON and 256 editing-OFF), ON/OFF editing RF pulses were centered at 1.9/7.5 ppm, and editing RF width = 14.4 ms. To facilitate internal tissue water referencing, unsuppressed water averages were acquired prior to the water-suppressed scans.

### Structural Imaging, Voxel Co-registration and Tissue Segmentation

Structural images were acquired at 3T, using 3D fast spoiled gradient-echo imaging (FSPGR) (Low et al., 1993) with the following parameters: TI = 650 ms, TE = 3 ms, TR = 6.7 ms, flip angle = 8^*o*^, FOV = 256 × 256 mm, resulting voxel size = 0.9 mm isotropic without gap, and scan time of ∼5 min. High-resolution T_1_ images were acquired sagittally and reformatted to axial and coronal oblique images parallel to the anterior commissure - posterior commissure (AC-PC) line. To ensure consistent voxel positioning in the left DLPFC, guidelines were to place the voxel on a double oblique image parallel to and between the superior and inferior frontal gyrus; these instructions were followed for all acquired scans. Grey matter (GM), white matter (WM), and cerebrospinal fluid (CSF) composition within the single voxel MEGA-PRESS data were determined using the GannetCoRegister module in Gannet (Harris et al., 2015) and FSL-FMRIB’s Automatic Segmentation Tool (FAST) (Zhang et al., 2001). Briefly, raw MRS data acquisition parameters (voxel size, orientation, and location stored in the MRS raw data headers) were determined to create a binary mask of the voxel locations, and co-registered to the T1-weighted images. This mask was then applied using FSL-FAST to determine GM/WM/CSF fractions (Figure 1B, Tables 1, 2, and Supplementary Table 4). Water-scaled metabolite concentrations were corrected for voxel tissue composition; observed metabolite concentrations (not corrected for metabolite relaxation times) were obtained, relative to the fully relaxed water concentration in tissue [M] by accounting for the volume fractions, water relaxation times (T1, T2) and water concentrations of the WM, GM, and CSF compartments, as per Gasparovic et al. (2006). Equations used for metabolite correction, and water relaxation times of the tissue compartments, are detailed in the Supplementary Methods.

### Magnetic Resonance Spectroscopy Data Processing

The Gannet 3.0 (Edden et al., 2014) processing pipeline was used to perform the following processing steps: frequency and phase correction by spectral registration, exponential line broadening (3 Hz) and a fast Fourier transform. The full-width at half-maximum (FWHM) of the modeled water signal was used to obtain the linewidth of the water reference. Once the OFF was subtracted from the ON acquisitions, a single reliable GABA+ peak at 3.01 ppm (representing the GABA peak co-edited with macromolecule [MM] signal), and a Glx doublet (co-edited glutamine + glutamate) peak at ∼3.75 ppm were fitted. Gaussian line shape fitting, with modifications to obtain GABA+ area outputs, were performed as in prior publications (Dubin et al., 2016; Da Silva et al., 2019). GABA+ and Glx levels were normalized by the area of the water model peak. An example spectra is provided in Figure 1C, and all (overlaid) spectra are provided in Supplementary Figure 1. GABA+ /Glx ratios were calculated to evaluate the inhibition/excitation balance within the voxel. The editing OFF acquisition was also processed using the FID-A toolkit (Simpson et al., 2017), which combined the receiver coil data, removed averages with significant motion, and performed spectral registration to frequency and phase correct the data prior to separating and combining the editing OFF averages. This output was then analyzed using the LCModel (version 6.3-0E) (Provencher, 1993) over the frequency range of 0.2–4.0 ppm. Metabolite reference spectra were generated using GAMMA library (Smith et al., 1994). The reference basis set consisted of 19 metabolites, as detailed in Supplementary Methods, and the corrected metabolite pseudo concentrations are shown in Supplementary Table 5.

### Quality Control

Seventy nine MRS scans from 53 participants (33 ASD; 20 control) were assessed for quality control. Reasons and details for scan exclusion (*n* = 3 baseline, *n* = 5 post-rTMS) are provided in Supplementary Table 1. We note that useable MRS data was not available for all rTMS trial participants due to imaging time-constraints, difficulties with scanning a complex clinical population, and due to our adherence to rigorous quality control of MRS data.

### Statistical Analysis

#### Analysis of Baseline Metabolite Levels in Autism Spectrum Disorder vs. Typically Developing Controls

Demographic variables, tissue composition and metabolites were compared between participants with ASD and TDCs. Tissue composition (GM, WM, and CSF fraction) and baseline metabolites (GABA+, Glx and GABA+ /Glx ratio) were compared between the ASD and TDC groups using a series of ANCOVAs, including age as a covariate. Group differences in NAA, GPC, mI, Glu, and Cr were also assessed.

#### Analysis of Magnetic Resonance Spectroscopy-Derived Metabolites at Pre- vs. Post-repetitive Transcranial Magnetic Stimulation in Clinical Trial Participants With Autism Spectrum Disorder

The effect of rTMS (active vs. sham) on metabolites was assessed in participants with ASD that completed the rTMS clinical trial. Pre/post-rTMS tissue composition (GM, WM, and CSF fractions) and metabolites (GABA+, Glx, and GABA+/Glx) were each compared between active and sham groups using 2 × 2 mixed-effect ANCOVAs, with rTMS group (active vs. sham rTMS) as a between-subjects factor, time (pre- vs. post-rTMS) as a within-subjects factor, and age as a covariate. Participants with MRS available at a single time point were included in these models, as single data points contribute to the overall group mean effects (cohort detailed in Table 2). To capture change in neurometabolite levels, irrespective of direction, the absolute value change for each metabolite (GABA+ and Glx levels, and GABA+ /Glx ratio) from pre- to post-rTMS was compared between active vs. sham groups, using ANCOVAs including age as a covariate. Only participants with MRS data available at both time points were included in these analyses (Supplementary Table 4 cohort). For metabolites that demonstrated change following rTMS, exploratory regressions were performed to test whether rTMS group moderated the relation between metabolite level change and baseline metabolite level. Simple effects of this moderation were tested using Aiken and West method (Aiken and West, 1991).

Baseline and pre/post analyses yielding significant p-values were corrected for multiple comparisons using the Benjamini Hochberg procedure (Benjamini and Hochberg, 1995), where appropriate (e.g., across GABA+, Glx and GABA+/Glx ratio analyses), and effect sizes were calculated.

## Results

### Analysis of Baseline Metabolite Levels in Autism Spectrum Disorder vs. Typically Developing Controls

Autism spectrum disorder and TDC participants had comparable demographic characteristics and voxel tissue composition, as detailed in Table 1. We did not find a significant effect of diagnostic group for metabolites at baseline; ASD and TDC participants did not differ in GABA+, Glx, or the GABA+/Glx ratio [all *F*_(__1,__44__)_ < 0.60, all *p* > 0.05] (Table 1). No diagnostic group differences were observed for NAA, GPC, mI, Glu, and Cr [all *F*_(__1,__44__)_ < 3.21, all *p* > 0.05] (Supplementary Table 5).

### Analysis of Magnetic Resonance Spectroscopy-Derived Metabolites Pre- vs. Post-repetitive Transcranial Magnetic Stimulation in Clinical Trial Participants With Autism Spectrum Disorder

Autism spectrum disorder participants in the active vs. sham rTMS groups with usable MRS data were found to have comparable demographic characteristics, as detailed in Table 2. However, participants with usable MRS data in the active group featured increased comorbidity on the MINI (*p* = 0.04, Fisher’s Exact), and were more often taking psychotropic medications (*p* < 0.001, Fisher’s Exact). Tissue fractions were comparable between active and sham rTMS groups, and over time there was no main effect of rTMS group for WM [*F*_(__1,__25__)_ = 0.25, *p* = 0.62], GM [*F*_(__1,__25__)_ = 0.13, *p* = 0.73] or CSF [*F*_(__1,__25__)_ = 0.16, *p* = 0.69], and no interaction between group and time, for WM [*F*_(__1,__25__)_ = 0.09, *p* = 0.77], GM [*F*_(__1,__25__)_ = 0.11, *p* = 0.75], or CSF [*F*_(__1,__25__)_ = 0.07, *p* = 0.79] fractions (Table 2).

**FIGURE 2:**
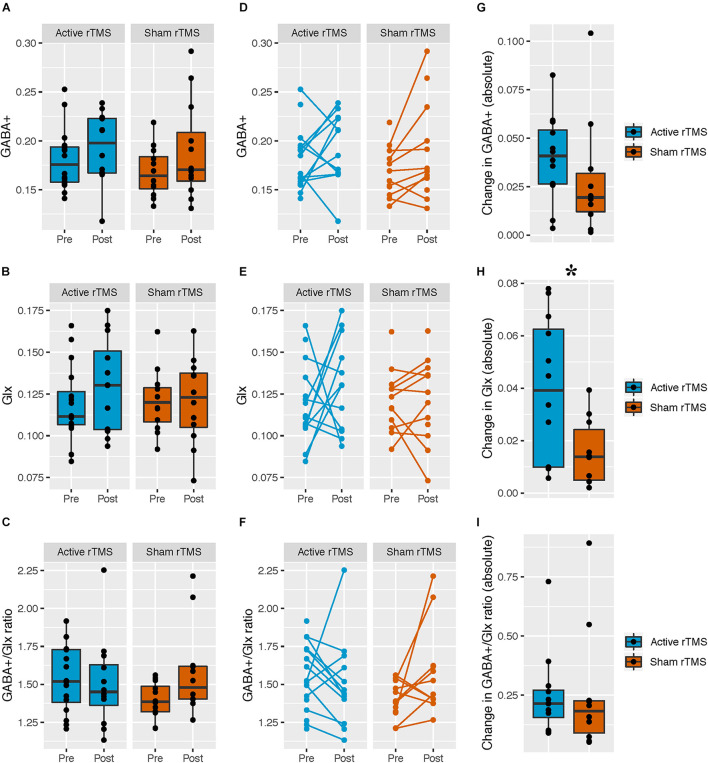
Pre- and post-rTMS GABA+, Glx, and GABA+/Glx ratio, in the left dorsolateral prefrontal cortex (DLPFC). **(A–C)** Levels of pre- and post-rTMS metabolites (GABA+, Glx) and the GABA+/Glx ratio, in the full ASD sample with MRS data; active rTMS (pre: *n* = 16; post: *n* = 12), sham rTMS (pre: *n* = 12; post: *n* = 12). Black lines denote the group medians. **(D–F)** Each participant is indicated by a pre-/post-rTMS pair of points, connected by a line. Unconnected points are from participants with MRS scans at a single time point. Active rTMS (pre: *n* = 16; post: *n* = 12), sham rTMS (pre: *n* = 12; post: *n* = 12). **(G–I)** The absolute value of the change in metabolites from pre- to post-rTMS in the ASD sample with matched pre- and post-rTMS scans only; active rTMS (*n* = 12), sham rTMS (*n* = 10). Black lines denote the group medians. *The absolute value change in Glx level was greater in the active compared to the sham rTMS group [*F*_(1,__19)_ = 6.54, *p* = 0.02].

Mean GABA+ and Glx levels, and the GABA+/Glx ratio did not differ from pre- to post-rTMS in either group; there was no main effect of rTMS group for GABA+ [*F*_(__1,__26__)_ = 0.33, *p* = 0.57], Glx [*F*_(__1,__25__)_ = 0.23, *p* = 0.64], or GABA+/Glx ratio [*F*_(__1,__25__)_ = 0.38, *p* = 0.55], no main effect of time for GABA+ [*F*_(__1,__24__)_ = 3.37, *p* = 0.08], Glx [*F*_(__1,__26__)_ = 0.72, *p* = 0.40], or GABA+/Glx ratio [*F*_(__1,__25__)_ = 0.84, *p* = 0.37], and no interaction between rTMS group and time, for GABA+ [*F*_(__1,__24__)_ = 0.46, *p* = 0.50], Glx [*F*_(__1,__26__)_ = 0.72, *p* = 0.40], or GABA+/Glx ratio [*F*_(__1,__25__)_ = 3.71, *p* = 0.07] (Table 2 and Figures 2A–C). Individual participant data are also shown as pre-/post-rTMS values (Figures 2D–F).

The absolute change in Glx level was greater in the active vs. sham rTMS group [*F*_(__1,__19__)_ = 6.54, *p* = 0.02 (FDR_*corr*_ *p* = 0.06), Cohen’s *f* = 0.59], whereas rTMS groups did not differ on change in absolute GABA+ level [*F*_(__1,__19__)_ = 0.89, *p* = 0.36] or GABA+/Glx ratio [*F*_(__1,__19__)_ = 0.005, *p* = 0.94] (Figures 2G–I). Given the apparent unequal variances between our absolute value Glx change data, we performed a Levene’s test to assess homogeneity of variance across active/sham groups, confirming the variances differed between groups [*F*_(__1,__20__)_ = 9.68, *p* < 0.05]. Welch’s *t*-test was then used to test for between-group differences, as appropriate when variances differ between groups; absolute Glx level change remained significantly different between active/sham groups [*t*_(__16__)_ = 16.22, *p* = 0.02]. To further confirm our results, we log-transformed our absolute value Glx change data, and confirmed the Levene’s test was not significant for our transformed data [*F*_(__1,__20__)_ = 0.04, *p* = 0.85] before proceeding. We then re-ran our original analysis using the log-transformed data (in order to include age as a covariate as per our original analysis), and found absolute Glx level change remained significantly different between active/sham groups [*F*_(__1,__19__)_ = 6.2, *p* = 0.02].

**FIGURE 3:**
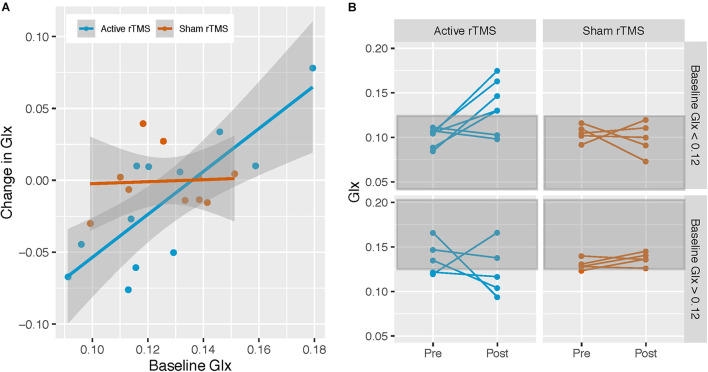
Active rTMS modulates Glx levels. **(A)** Associations between Glx levels at baseline, and change in Glx from pre- to post-rTMS in the left dorsolateral prefrontal cortex (DLPFC), in the active and sham rTMS groups; active rTMS (*n* = 12), sham rTMS (*n* = 10). Baseline Glx was associated with Glx change in the active rTMS group only: *b* = 1.49, *SE* = 0.31, *t*_(18)_ = 4.77, *p* < 0.001. **(B)** To visually demonstrate that Glx level increased in participants with lower baseline Glx levels, and that Glx level decreased in participants with higher baseline Glx levels, in the active rTMS group only, participants were stratified according to the median split of the baseline Glx level (median Glx level for entire ASD sample = 0.12) and their pre-/post-rTMS pair of points were plotted.

Repetitive transcranial magnetic stimulation group significantly moderated the relationship between baseline Glx and pre/post-rTMS Glx change [*F*_(__1,__17__)_ = 4.78, *p* = 0.04, Cohen’s *f* = 0.53] (Figure 3A). Simple effects analysis (Aiken and West, 1991) revealed that baseline Glx predicted pre/post-rTMS Glx change in the active [*b* = 1.52, *SE* = 0.32, *t*_(__17__)_ = 4.74, *p* < 0.001] but not the sham rTMS group [*b* = 0.13, *SE* = 0.55, *t*_(__17__)_ = 0.24, *p* = 0.81].

For visualization purposes, participants were divided according to the baseline Glx level by median split (median Glx level across all ASD participants = 0.12), and pre/post-rTMS Glx level for each participant was plotted. Participants in the active rTMS group whose baseline Glx levels were below the median had post-rTMS Glx levels that were higher, whereas participants whose baseline Glx levels were above the median had post-rTMS Glx levels that were similar or lower (Figure 3B). In contrast, post-rTMS Glx levels for participants in the sham rTMS group remained similar to their baseline Glx levels (Figure 3B).

Given that the DLPFC was stimulated bilaterally, yet MRS was acquired unilaterally, significant analyses were re-analyzed, adjusting for stimulation site order instead of age. Results remained unchanged; the absolute change in Glx level was greater in the active vs. sham rTMS group [*F*_(__1,__19__)_ = 6.46, *p* = 0.02 (FDR_*corr*_ *p* = 0.06), Cohen’s *f* = 0.58]. rTMS group significantly moderated the relationship between baseline Glx and pre/post-rTMS Glx change [*F*_(__1,__17__)_ = 4.97, *p* = 0.04, Cohen’s *f* = 0.54], and simple effects analysis revealed that baseline Glx predicted pre/post-rTMS Glx change in the active [*b* = 1.50, *SE* = 0.37, *t*_(__17__)_ = 4.02, *p* < 0.001] but not the sham-rTMS group [*b* = 0.07, *SE* = 0.56, *t*_(__17__)_ = 0.14, *p* = 0.89].

Although no formal analyses were undertaken to assess corresponding behavior changes with metabolite level change, qualitative data relating pre-post rTMS Glx level change to change in executive function outcome measures are provided in Supplementary Figure 2.

## Discussion

Using MRS, we compared GABA+ and Glx levels in young adults with ASD and clinically significant executive function deficits, prior to (baseline) and following their participation in a pilot randomized, double-blind, sham-controlled rTMS trial (20 sessions of active vs. sham rTMS to DLPFC) that tested the feasibility and preliminary efficacy of rTMS for the treatment of executive function deficits. Baseline metabolite levels from the entire ASD group were also compared to a TDC group with data available for the same baseline time-point. Our results suggest that while levels of GABA+, Glx, and their ratio, in the left DLPFC, may not differ in emerging adults with vs. without ASD, active rTMS can modulate Glx levels in individuals with ASD, and that the direction of change is associated with baseline Glx levels. Our results build on prior evidence in non-ASD samples that MRS appears to be sensitive to changes in cortical metabolism following rTMS.

Our findings based on the comparison of neurometabolite levels at baseline in ASD vs. TDC align with several studies, particularly in adult samples, that did not detect GABA + and/or Glx differences between participants with ASD vs. TDCs in prefrontal brain regions [i.e., the left DLPFC (Horder et al., 2013; Endres et al., 2017), right DLPFC (Kirkovski et al., 2018), dorsomedial prefrontal cortex (Ajram et al., 2017; Pretzsch et al., 2019a), and medial prefrontal cortex (Aoki et al., 2012; Carvalho Pereira et al., 2018)]. In contrast, our findings do not align with two studies conducted in children, which report reduced GABA+ and/or Glx levels in the frontal lobe of ASD vs. TDC participants (Harada et al., 2011; Kubas et al., 2012), suggesting that alterations could be more pronounced at the diagnostic group level earlier in development. GABA is critical for the functional maturation of the central nervous system, and dysfunctional GABA is thought to play a role in multiple neurodevelopmental disorders, including ASD (Smith-Hicks, 2013). Thus, even if GABA levels normalize by adulthood in individuals with ASD, it remains possible that the presence of altered GABA levels during childhood could contribute to atypical neurodevelopment. Moreover, aberrant excitatory-inhibitory neurotransmission in ASD may not manifest as uniformly higher or lower metabolite levels, which could conceal group-wise ASD-TDC differences. Interestingly, associations between GABA+ levels and scores on the Autism Spectrum Questionnaire (Brix et al., 2015) and Autism Diagnostic Interview–Revised (Carvalho Pereira et al., 2018) have been reported, despite the absence of between group (ASD vs. TDC) GABA+ level differences. Therefore, the capacity to alter metabolite levels within the GABA/glutamate neurotransmitter pathways may remain an important therapeutic target in individuals with ASD, irrespective of the presence or absence of ASD-TDC group differences.

In the current study, we found increased Glx level change following active but not sham rTMS. Our findings align with a number of prior MRS studies, conducted across healthy, depressed and schizophrenia samples, that demonstrate excitatory rTMS to left DLPFC alters the glutamatergic system (Michael et al., 2003; Luborzewski et al., 2007; Croarkin et al., 2016; Dlabac-de Lange et al., 2017). In our clinical trial sample of ASD participants with executive function impairment, Glx level change following active rTMS was associated with baseline Glx levels. A recent MRS study in participants with ASD also found that Glx level change following an intervention with cannabivarin (CBDV) (a cannabinoid compound) was associated with baseline Glx level in the basal ganglia (Pretzsch et al., 2019b). Moreover, neither our rTMS intervention, nor the CBVD intervention (Pretzsch et al., 2019b) had any impact on GABA+ levels in ASD. GABA, glutamate and glutamine are constantly in flux, and the final conversion to GABA is dependent upon the GAD enzyme. Within the GABA/glutamate metabolic pathways, Glx level change may be achieved more readily, as both glutamate and glutamine exist earlier along the conversion chain, and the enzyme required for the final conversion to GABA may be altered in some individuals with ASD (Yip et al., 2007). However, our findings may not be specific to ASD (or individuals with ASD and executive function impairments). Namely, a study conducted in healthy participants found that active (20 Hz) but not sham rTMS to left DLPFC increased Glx in the cingulate cortex, and that increases were most prominent in participants with lower baseline Glx (Michael et al., 2003). The observed modulation of Glx following active rTMS found here aligns with the concept of homeostatic plasticity (Daskalakis et al., 2006). Given that rTMS modifies brain physiology, it follows that its effect would depend upon an individual’s unique physiology during stimulation. In light of evidence that unilateral rTMS can alter contralateral cortical excitability (Plewnia et al., 2003), stimulation site order could conceivably induce different physiological effects in each hemisphere; however, our findings remained unchanged when we controlled for stimulation site order. Moreover, while unilateral (left DLPFC) stimulation is more common in the literature (Michael et al., 2003; Luborzewski et al., 2007; Zheng et al., 2010; Croarkin et al., 2016; Dubin et al., 2016; Baeken et al., 2017; Levitt et al., 2019), there is prior evidence that bilateral stimulation to DLPFC increases Glx levels in the left DLPFC of individuals with schizophrenia (Dlabac-de Lange et al., 2017). However, as with our study, Dlabac-de Lange et al. (2017) did not collect MRS data from the right DLPFC, despite stimulating the DLPFC bilaterally.

Some limitations to the present study warrant mention. First, our pilot clinical trial sample size is limited and usable MRS data for the rTMS intervention group was available for 28/40 participants from the full clinical trial. Pre/post MRS data was further limited to 22/40 clinical trial participants. We note that our sample size was smaller than anticipated due to the challenges of collecting pre/post imaging data in a complex clinical sample and based on our adherence to rigorous quality control of MRS data (which we consider a relative strength of our study). The published study from our pilot clinical trial found that participants with ASD and executive function deficits that also had lower adaptive (everyday) functioning exhibited improvements in spatial working memory following active rTMS (Ameis et al., 2020). Though we had hoped to relate metabolite change with behavior, owing to the sample size of participants with complete pre/post MRS data, we did not undertake statistical analyses to evaluate the relations between metabolites, cognition, and behavior due to concerns that the sample is underpowered to undertake such analyses and multiple testing may contribute to spurious findings. Therefore, the clinical meaningfulness of our presented findings remains unclear. While our findings are promising and align with previous evidence of excitatory rTMS effects on Glx, our results in ASD must be considered preliminary and are in need of replication in a larger sample with the opportunity to examine relationships with clinical outcomes. Second, our findings may not be broadly generalizable. Specifically, this study included emerging adults with ASD with clinically significant executive function impairments, thus our findings may not be generalizable to individuals across the autism spectrum. Relatedly, as the TDC group did not receive rTMS, we were not able to test whether rTMS-induced modulation of Glx is unique to our ASD sample. However, rTMS effects on Glx have been reported previously in non-ASD samples (Michael et al., 2003). Moreover, concurrent medication in the ASD groups may have affected GABA+ and Glx levels, though sample size constraints precluded investigations of medication effects. It would be valuable for future studies to compare metabolite levels across ASD and clinical samples taking similar medications (e.g., stimulants, SSRIs). We chose not to covary for medication in our analyses, as medications used were highly heterogeneous, with potential for variable effects on the excitation-inhibition system. Further, we did not collect race/ethnicity data as part of our clinical trial. We primarily recruited participants from a publicly funded mental health clinic at our Center with specific policies in place to ensure equitable access to care across the city of Toronto. We therefore expect our sample would be broadly in line with the diverse composition of the city of Toronto. Third, neurometabolites were evaluated from a single voxel in the left DLPFC, yet rTMS was administered to bilateral DLPFC in a sequential order throughout the clinical trial. Future bilateral rTMS studies in ASD should acquire MRS data bilaterally to help tease apart potential hemispheric differences in neurophysiological effects following rTMS. Fourth, the relative amounts of glutamine and glutamate that contribute to the Glx signal could not be differentiated with the MEGA-PRESS sequence used. However, it is likely that the Glx signal predominantly reflects glutamate as glutamate is present in higher concentrations than glutamine in the brain, and glutamine can be below the detection limit of MRS (e.g., <1 mM) (Hancu and Port, 2011). The stability of water-referenced GABA and Glx using MEGA-PRESS has been demonstrated in the same individual over a 3-month period (Ferland et al., 2019), and a large multi-site study demonstrated that water-referenced GABA is a viable and reliable method to quantify GABA levels *in vivo* (Mikkelsen et al., 2019). Of note, GABA+ measurements reflect GABA plus underlying macromolecules, and it is unknown if/how macromolecules are altered in pathology. Fifth, metabolite levels are typically assessed within 24 h post-rTMS, though Glx levels in depressed adolescents have been shown to increase for up to 6-months post stimulation (Croarkin et al., 2016). Thus, we may not have captured the full extent of metabolite level change induced by rTMS, and future studies including a longer follow-up period will be required to clarify this. Lastly, due to the small number of females included in our sample, we were unable to assess how sex/gender modulate the present findings. Notably, the active rTMS group had a larger (though non-significant) proportion of females than the sham rTMS group. The published study from this pilot clinical trial found an interaction effect between rTMS group, time and sex on executive functioning, such that executive functioning improved to a greater extent in females in the active vs. sham group (Ameis et al., 2020). Recent neuroimaging work suggests that imbalanced excitation-inhibition within social-cognitive brain regions may be more pronounced in males vs. females with ASD (Trakoshis et al., 2020). Thus, future clinical trials should consider sex/gender, when possible. Future trials should also consider relations between neurometabolite levels and depressive symptoms in young adults with ASD, especially given recent preliminary evidence that rTMS to the DLPFC may improve depressive symptoms in adults with ASD (Gwynette et al., 2020).

Given that modulation of corticospinal excitability is thought to involve glutamatergic and/or GABAergic receptor pathways (Huang et al., 2007; Stagg et al., 2009), it is promising that we found rTMS to be a useful probe and modulator of the glutamatergic system in individuals with ASD. The current finding that rTMS yields a change in Glx that is measurable with MRS builds on prior similar findings in non-ASD samples and is encouraging for future studies aimed at better understanding the mechanism of action of rTMS in the service of harnessing its interventional potential. Uncovering how baseline metabolite levels relate to metabolite level change and clinical outcomes across different clinical populations could meaningfully inform future rTMS study designs in ASD and beyond.

## Data Availability Statement

The datasets presented in this article are not readily available because we do not have consent to share this data.

## Ethics Statement

The studies involving human participants were reviewed and approved by The Centre for Addiction and Mental Health (CAMH) Research Ethics Board (REB; protocol #119-2013). This study was registered with Clinicaltrials.gov (ID: NCT02311751). All participants provided written informed consent to participate in the current study. Written informed consent from the participants’ legal guardian/next of kin was not required to participate in this study in accordance with the national legislation and the institutional requirements.

## Author Contributions

SHA, PS, ZJD, DMB, PD, NS, M-CL, PEC and IM-E contributed to the design of this work. REL, M-CL, and SHA collected the data. PT and PS advised on the MRS analyses. IM-E, NJF, and HT analyzed the data. IM-E and SHA interpreted the data and drafted this work. All the authors reviewed, edited, and approved a final version.

## Conflict of Interest

ZJD received research support and in-kind equipment support for an investigator-initiated study from Brainsway Ltd. He has also received in-kind equipment support from Magventure for investigator-initiated research. DMB received research support and in-kind equipment support for an investigator-initiated study from Brainsway Ltd., and he is the site principal investigator for three sponsor-initiated studies for Brainsway Ltd. He receives in-kind equipment support from Magventure for investigator-initiated research. He received medication supplies for an investigator-initiated trial from Indivior. He has participated in an advisory board for Janssen. PEC has received research grant support from Pfizer Inc.; equipment support from Neuronetics Inc.; and received supplies and genotyping services from Assurex Health Inc., for investigator-initiated studies. He is the primary investigator for a multicenter study funded by Neuronetics Inc., and a site primary investigator for a study funded by NeoSync Inc. He has served as a paid consultant for Procter & Gamble Company and Myriad Neuroscience. The remaining authors declare that the research was conducted in the absence of any commercial or financial relationships that could be construed as a potential conflict of interest.

## Publisher’s Note

All claims expressed in this article are solely those of the authors and do not necessarily represent those of their affiliated organizations, or those of the publisher, the editors and the reviewers. Any product that may be evaluated in this article, or claim that may be made by its manufacturer, is not guaranteed or endorsed by the publisher.
