# Supplementary material for: Modulation of Dorsolateral Prefrontal Cortex Glutamate/Glutamine Levels Following Repetitive Transcranial Magnetic Stimulation in Young Adults With Autism
Source: Front Neurosci. 2021 Oct 6;15:711542. doi: 10.3389/fnins.2021.711542 (PMC8527173; doi:10.3389/fnins.2021.711542)
Supplement: Supplementary file 1 [file Data_Sheet_1.pdf]

## Supplementary Table 1

| | TDC | ASD: Active rTMS | | ASD: Sham rTMS | |
|---|---|---|---|---|---|
| **Original N** | 20 | 20 | | 20 | |
| **MRS scans** | | Pre (Baseline) | Post | Pre (Baseline) | Post |
| Missing both timepoints | | 3 | | 4 | |
| Missing single timepoint | - | - | 2 * | 3 | 2 |
| **Assessed for quality control** | 20 | 17 | 15 | 13 | 14 |
| **Excluded during quality control** | 1<br>Linewidth >10 | 1<br>Linewidth >10 | 3<br>Linewidth >10<br>Linewidth >10<br>Incorrect voxel placement | 1<br>Linewidth >10 | 2<br>Excess motion<br>Excess motion |
| **Total N** | 19 | 16 | 12 | 12 | 12 |
| **Baseline** | 19 | 28 | | | |

**rTMS clinical trial**

| | Active Pre | Active Post | Sham Pre | Sham Post |
|---|---|---|---|---|
| ASD participants with useable scans (pre and/or post) | 16 | 12 | 12 | 12 |
| ASD participants with matched pre and post scans | 12 | 12 | 10 | 10 |

* Both participants dropped out of the trial after 1-2 rTMS sessions.

TDC: typically developing controls; ASD: autism spectrum disorder; rTMS: repetitive transcranial magnetic stimulation; MRS: magnetic resonance spectroscopy

Scans were excluded for the following reasons: excess motion (n=2), incorrect voxel placement (n=1), linewidth > 10 Hz (n=5). Linewidth represents the homogeneity of the magnetic field within the voxel, and values above 10 could yield inaccurate GABA + results.

Mean (SD) linewidth for the *baseline cohort*: TDC = 8.68 (0.80), ASD = 8.71 (0.94) ($t = 0.12$, $p = 0.91$), and for the *ASD treatment cohort (rTMS clinical trial)*: Active rTMS: 9.00 (0.73), Sham rTMS = 8.33 (1.07) ($F = 6.51$, $p = 0.02$; linewidth was higher in the active group).

Mean (SD) signal to noise ratio (SNR) for the *baseline cohort*: TDC = 44.8 (3.81), ASD = 41.5 (6.58) ($t = -1.98$, $p = 0.05$; SNR was marginally higher in the TDC group), and for the *ASD treatment cohort (rTMS clinical trial)*: Active rTMS: 41.5 (4.40), Sham rTMS = 41.4 (8.94) ($F = 0.03$, $p = 0.86$).

**Supplementary Table 1.** Participant flow: indicating the number of participants enrolled in the clinical trial, the MRS data that was missing or excluded (reasons provided), and final numbers for participants included in the analyses. Linewidth and SNR for the baseline and ASD treatment cohorts are detailed.

## Supplementary Table 2

| | Included ASD participants (n=28) | Excluded ASD participants (n=12) | p value |
|---|---|---|---|
| **Age** | | | |
| Mean (SD) | 21.0 (3.67) | 23.3 (4.69) | 0.15 |
| Median [Min, Max] | 21.0 [16.0, 27.0] | 22.0 [16.0, 33.0] | |
| **Sex** | | | |
| Number of males (%) | 7 (58.3%) | 21 (75.0%) | 0.50 |
| **Psychotropic Medication*** | | | |
| Number of participants on (%) | 9 (75.0%) | 17 (60.7%) | 0.61 |
| **MINI** | | | |
| **Comorbidity** | | | |
| Number of participants (%) | 9 (75.0%) | 16 (57.1%) | 0.48 |
| **Depression - current (2 weeks)** | | | |
| Number of participants (%) | 3 (25.0%) | 7 (25.0%) | 1.00 |
| **Depression - recurrent** | | | |
| Number of participants (%) | 3 (25.0%) | 2 (7.1%) | 0.27 |
| **Years of Education** | | | |
| Mean (SD) | 13.0 (2.26) | 14.2 (3.01) | 0.22 |
| Median [Min, Max] | 12.0 [10.0, 18.0] | 13.5 [10.0, 22.0] | |
| **IQ - General Abilities Index** | | | |
| Mean (SD) | 104 (16.2) | 112 (17.8) | 0.16 |
| Median [Min, Max] | 104 [81.0, 134] | 111 [77.0, 141] | |
| **BRIEF Metacognition Index** | | | |
| Mean (SD) | 74.2 (10.7) | 70.6 (8.21) | 0.26 |
| Median [Min, Max] | 76.5 [58.0, 88.0] | 68.5 [59.0, 84.0] | |
| **BRIEF Global Composite** | | | |
| Mean (SD) | 72.3 (11.1) | 68.0 (8.18) | 0.18 |
| Median [Min, Max] | 73.5 [53.0, 86.0] | 66.5 [52.0, 86.0] | |
| **Adaptive Functioning Composite** | | | |
| Mean (SD) | 72.0 (12.2) | 75.5 (9.88) | 0.34 |
| Median [Min, Max] | 75.5 [43.0, 92.0] | 74.5 [58.0, 104] | |

Participants were excluded if they did not have MRS data (n=10), or if their data was excluded at the quality control stage (n=2)
*Psychotropic medication is detailed in Supplementary Table 3 (for the included participants)
ASD: autism spectrum disorder; BRIEF: Behavior Rating Inventory of Executive Function; MINI: Mini International Neuropsychiatric Interview

**Supplementary Table 2.** Characteristics of the ASD participants with and without useable magnetic resonance spectroscopy (MRS) data at baseline. Original clinical trial sample (n=40): participants with useable MRS data were included in the present study (n=28), whereas participants without useable MRS data were excluded (n=12). The included and excluded participants did not differ in demographic or clinical characteristics.

## Supplementary Table 3

| Participants that endorsed the following (n) | Baseline Cohort | | rTMS Clinical Trial Cohort | |
|---|---|---|---|---|
| | TDC[a] (n=19) | ASD (n=28) | Active rTMS (n=16) | Sham rTMS (n=12) |
| **MINI - Comorbidity** | 0 (0%) | 16 (57.1%) | 10 (62.5%) | 6 (50.0%) |
| Major Depressive Episode | 0 (0%) | 7 (25.0%) | 2 (12.5%) | 5 (41.7%) |
| Suicidality | 0 (0%) | 13 (46.4%) | 7 (43.8%) | 6 (50.0%) |
| Hypomanic Episode | 0 (0%) | 3 (10.7%) | 2 (12.5%) | 1 (8.3%) |
| Panic Disorder | 0 (0%) | 2 (7.1%) | 2 (12.5%) | 0 (0%) |
| Agorophobia | 0 (0%) | 4 (14.3%) | 3 (18.8%) | 1 (8.3%) |
| Generalized Social Phobia | 0 (0%) | 2 (7.1%) | 0 (0%) | 2 (16.7%) |
| Obsessive Compulsive Disorder | 0 (0%) | 1 (3.6%) | 0 (0%) | 1 (8.3%) |
| Psychotic Disorders | 0 (0%) | 1 (3.6%) | 1 (6.2%) | 0 (0%) |
| Mood Disorder with Psychotic Features | 0 (0%) | 2 (7.1%) | 1 (6.2%) | 1 (8.3%) |
| Generalized Anxiety Disorder | 0 (0%) | 9 (32.1%) | 5 (31.2%) | 4 (33.3%) |
| Anorexia Nervosa | 0 (0%) | 1 (3.6%) | 0 (0%) | 1 (8.3%) |
| Antisocial Personality Disorder | 0 (0%) | 1 (3.6%) | 1 (6.2%) | 0 (0%) |
| **Participants taking the following psychoptripic medications (n)** | | | | |
| Selective-Serotonin Reuptake Inhibitor (SSRI) | 0 (0%) | 10 (35.7%) | 7 (43.8%) | 3 (25.0%) |
| Selective-Norepinepherine Reuptake Inhibitor (SNRI) | 0 (0%) | 1 (3.6%) | 0 (0%) | 1 (8.3%) |
| Tricyclic Antidepressant (TCA) | 0 (0%) | 0 (0%) | 0 (0%) | 0 (0%) |
| Tetracyclic Antidepressant | 0 (0%) | 1 (3.6%) | 1 (6.2%) | 0 (0%) |
| Norephinephrine-Dopamine Reuptake Inhibitor (NDRI) | 0 (0%) | 2 (7.1%) | 1 (6.2%) | 1 (8.3%) |
| Atypical Antipsychotic | 0 (0%) | 4 (14.3%) | 3 (18.8%) | 1 (8.3%) |
| Amphetamine | 0 (0%) | 1 (3.6%) | 1 (6.2%) | 0 (0%) |
| Methylphenidate | 0 (0%) | 4 (14.3%) | 4 (25.0%) | 0 (0%) |
| Benzodiazepine* | 0 (0%) | 2 (7.1%) | 1 (6.2%) | 1 (8.3%) |
| Medical Marijuana | 0 (0%) | 1 (3.6%) | 0 (0%) | 1 (8.3%) |

MINI: Mini International Neuropsychiatric Interview; TDC: typically developing controls; ASD: autism spectrum disorder; rTMS: repetitive transcranial magnetic stimulation

[a] Psychotropic medication data was missing for 1 TDC participant
*Participants on benzodiazepines were taking < 2mg lorazepam equivalent

**Supplementary Table 3**. Psychiatric comorbidities from the MINI, and psychotropic medication details, for the baseline and rTMS clinical trial samples.

## Supplementary Table 4

| | Active rTMS (n=12) | | Sham rTMS (n=10) | |
|---|---|---|---|---|
| **Age** | | | | |
| Mean (SD) | 24.0 (5.08) | | 24.2 (5.01) | |
| Median [Min, Max] | 24.0 [16.0, 33.0] | | 26.0 [16.0, 31.0] | |
| **Sex** | | | | |
| Number of males (%) | 9 (75.0%) | | 7 (70.0%) | |
| **Psychotropic Medication*** | | | | |
| Number of participants on (%) | 9 (75.0%) | | 3 (30.0%) | |
| **MINI** | | | | |
| **Comorbidity** | | | | |
| Number of participants (%) | 8 (66.7%) | | 5 (50.0%) | |
| **Depression - current (2 weeks)** | | | | |
| Number of participants (%) | 2 (16.7%) | | 4 (40.0%) | |
| **Depression - recurrent** | | | | |
| Number of participants (%) | 0 (0%) | | 2 (20.0%) | |
| **Years of Education** | | | | |
| Mean (SD) | 15.1 (3.55) | | 13.6 (2.84) | |
| Median [Min, Max] | 15.0 [10.0, 22.0] | | 12.5 [10.0, 18.0] | |
| **IQ - General Abilities Index** | | | | |
| Mean (SD) | 109 (18.1) | | 109 (14.7) | |
| Median [Min, Max] | 107 [77.0, 133] | | 107 [92.0, 141] | |
| **BRIEF Metacognition Index** | | | | |
| Mean (SD) | 71.3 (8.39) | | 70.7 (10.2) | |
| Median [Min, Max] | 70.0 [59.0, 84.0] | | 67.0 [59.0, 84.0] | |
| **BRIEF Global Composite** | | | | |
| Mean (SD) | 67.8 (8.77) | | 70.8 (8.64) | |
| Median [Min, Max] | 67.5 [52.0, 86.0] | | 67.0 [62.0, 83.0] | |
| **Adaptive Functioning Composite** | | | | |
| Mean (SD) | 75.3 (8.99) | | 73.0 (8.88) | |
| Median [Min, Max] | 75.5 [61.0, 89.0] | | 72.0 [58.0, 86.0] | |
| | **Pre (n=12)** | **Post (n=12)** | **Pre (n=10)** | **Post (n=10)** |
| **WM fraction** | | | | |
| Mean (SD) | 0.387 (0.0793) | 0.432 (0.105) | 0.374 (0.0654) | 0.394 (0.0916) |
| Median [Min, Max] | 0.397 [0.222, 0.500] | 0.423 [0.310, 0.600] | 0.379 [0.268, 0.463] | 0.383 [0.270, 0.537] |
| **GM fraction** | | | | |
| Mean (SD) | 0.463 (0.0404) | 0.438 (0.0729) | 0.472 (0.0380) | 0.457 (0.0581) |
| Median [Min, Max] | 0.465 [0.398, 0.530] | 0.450 [0.321, 0.542] | 0.466 [0.428, 0.557] | 0.452 [0.379, 0.546] |
| **CSF fraction** | | | | |
| Mean (SD) | 0.135 (0.0308) | 0.123 (0.0365) | 0.135 (0.0277) | 0.136 (0.0306) |
| Median [Min, Max] | 0.128 [0.0963, 0.208] | 0.117 [0.0794, 0.209] | 0.141 [0.0960, 0.166] | 0.151 [0.0793, 0.168] |
| **GABA+** | | | | |
| Mean (SD) | 0.181 (0.0292) | 0.193 (0.0364) | 0.164 (0.0215) | 0.186 (0.0476) |
| Median [Min, Max] | 0.176 [0.147, 0.253] | 0.198 [0.118, 0.239] | 0.164 [0.133, 0.195] | 0.170 [0.131, 0.292] |
| **Glx** | | | | |
| Mean (SD) | 0.116 (0.0233) | 0.130 (0.0281) | 0.117 (0.0151) | 0.118 (0.0238) |
| Median [Min, Max] | 0.109 [0.0846, 0.166] | 0.130 [0.0938, 0.175] | 0.120 [0.0919, 0.140] | 0.123 [0.0731, 0.145] |
| **GABA+/Glx ratio** | | | | |
| Mean (SD) | 1.57 (0.187) | 1.51 (0.297) | 1.41 (0.128) | 1.59 (0.312) |
| Median [Min, Max] | 1.57 [1.23, 1.81] | 1.45 [1.13, 2.25] | 1.42 [1.21, 1.56] | 1.48 [1.27, 2.21] |

* Psychotropic medication is detailed in Supplementary Table 3

rTMS: repetitive transcranial magnetic stimulation; MINI: Mini International Neuropsychiatric Interview; BRIEF: Behavior Rating Inventory of Executive Function; WM: white matter, GM: grey matter; CSF: cerebrospinal fluid

**Supplementary Table 4.** Characteristics of the clinical trial sample (active and sham rTMS), for participants with complete pre/post magnetic resonance spectroscopy (MRS) data only. This slightly smaller sample was used for analyses that required ASD participants to have both a pre- and a post-rTMS MRS scan (i.e., assessment of absolute value metabolite level change, and evaluating the association between baseline Glx and pre/post-rTMS Glx change).

## Supplementary Table 5

| | TDC (n=19) | ASD (n=28) | Test | p value |
|---|---|---|---|---|
| **Glutamate (Glu)** | | | | |
| Mean (SD) | 3.93 (0.470) | 3.58 (0.730) | $F_{(1,44)} = 3.20$ | 0.08 |
| Median [Min, Max] | 3.92 [3.11, 4.63] | 3.49 [2.46, 4.87] | | |
| **N-acetyl Acetate (NAA)** | | | | |
| Mean (SD) | 7.05 (1.23) | 6.53 (1.16) | $F_{(1,44)} = 1.97$ | 0.17 |
| Median [Min, Max] | 6.69 [5.36, 9.21] | 6.43 [4.47, 8.36] | | |
| **Creatine (Cr)** | | | | |
| Mean (SD) | 4.92 (0.613) | 4.67 (0.784) | $F_{(1,44)} = 1.27$ | 0.27 |
| Median [Min, Max] | 4.75 [3.81, 5.99] | 4.57 [3.56, 6.34] | | |
| **myo-Inositol (mI)** | | | | |
| Mean (SD) | 2.73 (0.415) | 2.73 (0.619) | $F_{(1,44)} < 0.001$ | 0.98 |
| Median [Min, Max] | 2.69 [2.05, 3.57] | 2.72 [1.77, 3.71] | | |
| **glycerolphophorylcholine (GPC)** | | | | |
| Mean (SD) | 1.12 (0.245) | 1.09 (0.298) | $F_{(1,44)} = 0.16$ | 0.69 |
| Median [Min, Max] | 1.11 [0.756, 1.50] | 1.07 [0.568, 1.70] | | |

Statistical tests represent groups comparisons, covarying for age
TDC: typically developing controls; ASD: autism spectrum disorder

**Supplementary Table 5.** LCModel metabolite values for ASD and TDC participants at baseline.

## Supplementary Methods

### A. ***Equations used for metabolite correction, and water relaxation times in each tissue compartment***

Water-scaled metabolite concentrations were corrected for voxel tissue composition using the following equation:

$$[M] = \frac{[M]_{ws}\left((f_{CSF} \times 55556 \times R_{CSF}) + (f_{GM} \times 43300 \times R_{GM}) + (f_{WM} \times 35580 \times R_{WM})\right)}{(1 - f_{CSF})}$$

The LCModel performs an operation to give water-scaled data (i.e., [M] WS); to reconcile this, (0.7*35880) was added to the denominator of this equation, to undo the assumptions used by the LCModel.

Water relaxation times for each tissue compartment (white matter [WM], grey matter [GM] and cerebrospinal fluid [CSF]) were calculated with the following equation:

$$R_i = \left(1 - e^{\left(-\frac{TR}{T1_i}\right)}\right) e^{\left(-\frac{TE}{T2_i}\right)}$$

Where *i* = WM, GM and CSF, resulting in the following equations:

$$R_{CSF} = \left(1 - e^{\left(-\frac{1500}{4000}\right)}\right) e^{\left(-\frac{68}{2000}\right)}$$

$$R_{GM} = \left(1 - e^{\left(-\frac{1500}{1200}\right)}\right) e^{\left(-\frac{68}{100}\right)}$$

$$R_{WM} = \left(1 - e^{\left(-\frac{1500}{800}\right)}\right) e^{\left(-\frac{68}{80}\right)}$$

### B. ***Basis set for LCModel fitting***

Reference basis set consisted of 19 metabolites: alanine (Ala), ascorbate (Asc), aspartate (Asp), creatine (Cr), gamma-aminobutyric acid (GABA), glucose (Glc), glutamine (Gln), glutamate (Glu), glutathione (GSH), glycerophosphocholine (GPC), lactate (Lac), myo-inositol (mI), *N*-acetylaspartate (NAA), *N*-acetylaspartylglutamate (NAAG), phosphocholine (PC), phosphocreatine (PCr), phosphoethanolamine (PE), scyllo-inositol (sI), and taurine (Tau). The quality of spectral fitting was assessed using the standard Cramer-Rao lower bound (CRLB) values for time-domain fitting and fitted spectra with CRLB < 20% accepted. Metabolite signal is attenuated due to the longer TE (68ms), compared to typical MRS scans TE (35ms), so only these major peaks are reliability fitted.

## Supplementary Figure 1

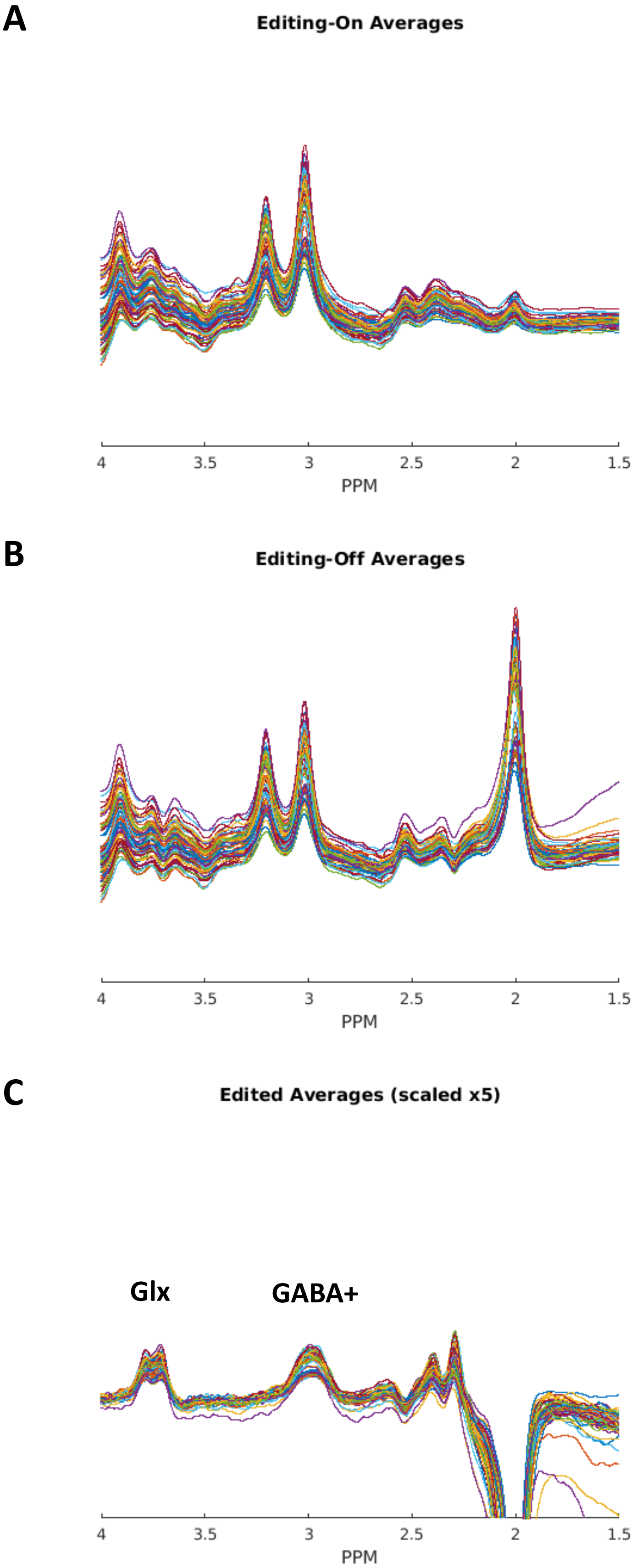


**Supplementary Figure 1.** Overlaid spectra from participants included in this study; datasets with poor shim or excessive subject motion were removed (as detailed in Supplementary Table 1). Subspectra acquired with the editing pulse on (**A**), off (**B**). Difference between on and off subspectra (**C**), showing edited GABA+ and Glx resonances.

## Supplementary Figure 2

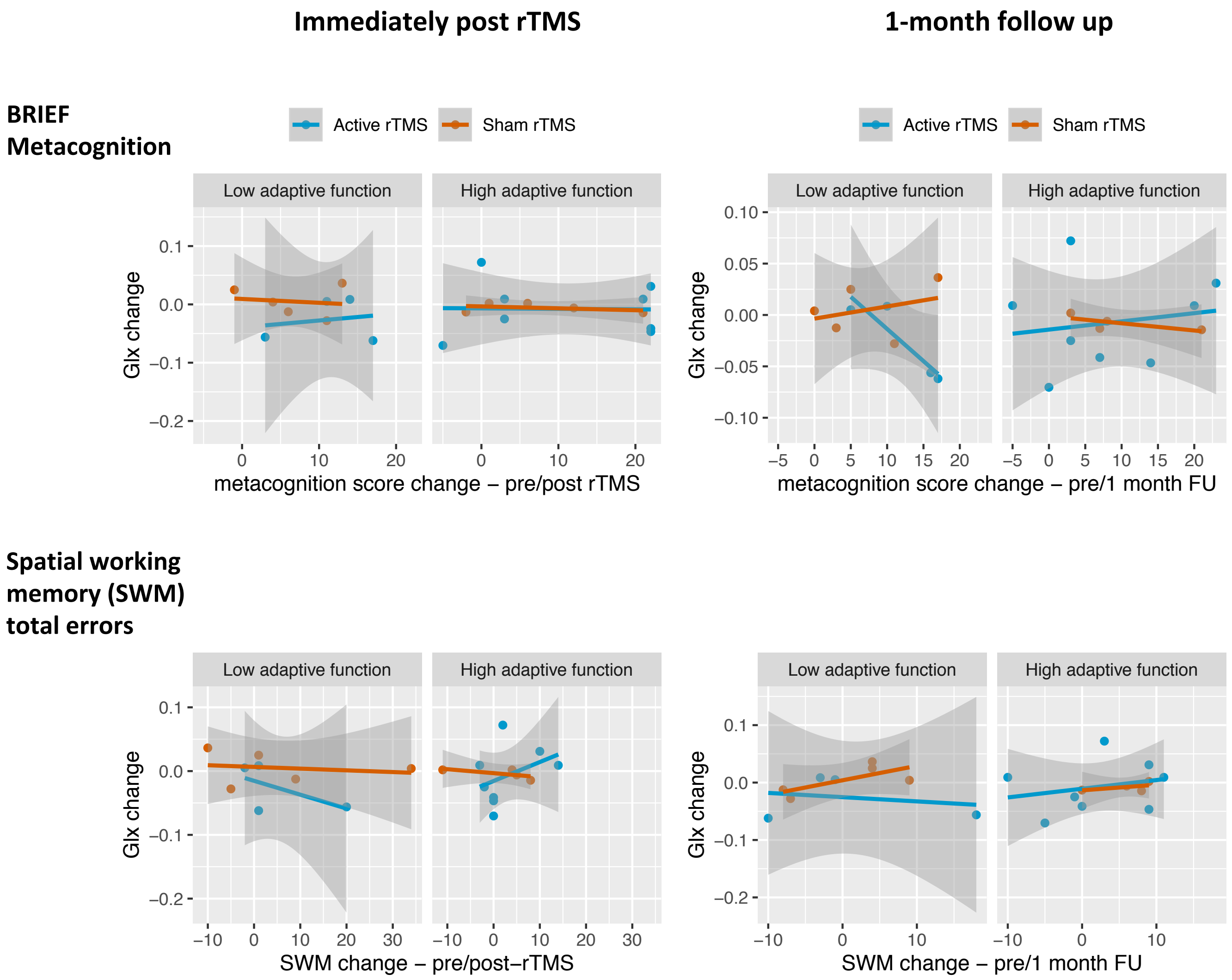


These behavioral data are provided as we recognize they may be of interest to some readers, based on the published findings from our pilot clinical trial (where we found that participants with ASD and executive function deficits that also had lower adaptive function exhibited improvements in spatial working memory following active rTMS [Ameis et al., 2020]). BRIEF: Behavior Rating Inventory of Executive Function.

No corresponding statistics were conducted, and no conclusions are drawn from these data.
